# Generic Health Literacy Measurements for Adults: A Scoping Review

**DOI:** 10.3390/ijerph17217768

**Published:** 2020-10-23

**Authors:** Cindy Yue Tian, Richard Huan Xu, Phoenix Kit-Han Mo, Dong Dong, Eliza Lai-Yi Wong

**Affiliations:** 1 Jockey Club School of Public Health and Primary Care, Faculty of Medicine, The Chinese University of Hong Kong, Hong Kong 999077, China; tianyue@link.cuhk.edu.hk (C.Y.T.); richardhxu@cuhk.edu.hk (R.H.X.); phoenix.mo@cuhk.edu.hk (P.K.-H.M.); dongdong@cuhk.edu.hk (D.D.); 2 Centre for Health Systems and Policy Research, Jockey Club School of Public Health and Primary Care, The Chinese University of Hong Kong, Hong Kong 999077, China

**Keywords:** generic health literacy, measurements, adults, social determinants of health

## Abstract

Background: Generic health literacy measurement (GHLM) is an important tool to identify individuals with limited health literacy and can assist the design of tailored interventions for improving public health literacy. However, there is no consensus on measuring generic health literacy. The present study aims to review current GHLM used for adults in the literature. Methods: A scoping review was undertaken to map the available measurements designed to assess generic health literacy. Results: The review identified 19 GHLM for adults. Most of them applied a multidimensional definition of health literacy with a focus on individuals’ abilities to access, appraise, understand, and apply health information and services. Nutbeam’s conceptual model and Sørensen’s integrated model were widely used among the identified measures as the theoretical foundation. While the social determinants of health (SDH) were acknowledged in the two models, it remains unmentioned in many of the identified measures based on the Nutbeam’s model and needs further development in the measure based on the Sørensen’s model. A total of 39 different domains were assessed in the 19 measurements: prose was identified in 8 measurements and was the most prominent domain; followed by numeracy (*n* = 7) and interactive (*n* = 7). SDH related domains such as social support (*n* = 3), social capital (*n* = 1) were seldom included in the identified measurements. Conclusions: Although current GHLM adopted a multidimensional construct, they mainly focused on individuals’ abilities and SDH has not been well-developed in the assessment. Further research is required to advance the measuring of the interaction between SDH and health literacy.

## 1. Introduction

Health literacy is an important public health agendum [1]. It is frequently defined as the ability of an individual to obtain, process, understand, and use health information and services to promote and maintain good health [2,3,4]. A large body of studies showed that inadequate health literacy has a strong impact on various health outcomes including lower utilization rate of preventive measures [5] and emergency services [6,7], higher hospitalization rate [6,7] and healthcare costs [8], health behavior [9], and health equity [10]. Health literacy is a state which can be improved through health education programs and practices. The fundamental step to enhance health literacy is to understand the current situation, and to design ways to improve it. Therefore, it is crucial for health workers, policymakers, and researchers to make health literacy quantifiable and comparable across different populations.

Instead of only focusing on one particular disease with relevant health issues, or a single channel to get health information, conducting a generic health literacy assessment is an essential way to identify people with limited health literacy and guide the design of tailored interventions to advance population health literacy. In this study, we focused on the generic health literacy measurement (GHLM), which refers to non-disease and non-domain specific health literacy instrument that assess population’s health literacy level. Many countries have conducted genetic health literacy surveys to evaluate and monitor adults’ health literacy levels. The results indicate that a health literacy crisis exists in developed countries and beyond. In 2003, the national representative assessment of health literacy among American adults showed that over one third of subjects had basic or below basic health literacy skills [11]. Later, a population-based health literacy survey conducted among adults using the same scale in Canada [12] and Australia [13] found that more than half of Canadians and Australians had limited health literacy. This crisis is even more obvious in developing countries. The national survey on health literacy in China in 2012 suggested that 91% of Chinese residents had insufficient knowledge about health [14].

However, as previous reviews indicated, there is no consensus on GHLM [15,16,17]. In the early days, health literacy measurements solely focused on the individuals’ ability to read and comprehend health-related materials in a clinical setting. For example, the United States’ health literacy measurement in 2003 only assessed the participant’s prose, document, and quantitative literacy [11]. In recent decades, a growing number of health literacy measures address a broader set of knowledge, skills, and attitudes needed to facilitate health decision-making outside of healthcare settings. Nutbeam highlighted that health literacy involved three types: functional (reading and writing skills to function effectively in an everyday situation), interactive (advanced cognitive and social skills to extract information from all kinds of form of communication), and critical (more advanced cognitive and social skills to handle information and have control over situations) [18]. Sørensen et al. considered the individual’s competency to get and apply health information and health care services was referenced across the domains of healthcare, disease prevention, and health promotion [19]. More recently, scholars tend to embrace the two-sided nature of health literacy. They argued that health literacy is not solely an individual characteristic, but is also involved system demands and complexities which can make the health information or services more accessible and accomplished to individual needs [20,21]. In this way, health literacy is not just about the individuals’ abilities, but also subject to social determinants of health (SDH) within the environment they embed. SDH are usually identified as the conditions in the environment in which people are born, live, and work that have impact on a wide range of health [22]. Many studies showed that appropriate interventions to address SDH can improve individual’s health literacy level and enable the individual to overcome barriers to health [9,23,24]. Therefore, the assessment of health literacy should not be solely considered from the individual level, but also from the social level with a focus on SDH.

Overall, a number of measurements examine generic health literacy using a range of indicators. The call for action to develop a reliable and valid measurement that allows international comparisons in health literacy research is receiving increasing attention [21,25]. To our knowledge, there are only two reviews on health literacy measurements exclusively targeting the adult population. The first report identified a total of 11 health literacy measures with a focus on the health-related fields in which these tools adopted and abilities that these tools measured [16]. The second focused on the theoretical foundation and limitation of the seven most frequently mentioned and cited health literacy measures [26]. In brief, the two studies acknowledged the complexity of health literacy measurements and the limited focus of current available measurements. However, both reviews overlooked the influence of SDH on health literacy, which is an important issue in this research area. In order to enhance the development of GHLM, a continuing discussion about the measurement of health literacy among adults is still needed.

From these perspectives, we are keen to review and synthesize current GHLM and to guide the future development and validation of a new measure. That promoted the necessity of conducting a scoping review to map existing GHLM. Scoping review is a relatively new approach to describe collected information and to answer to a broad question. In the present study, we aim to retrieve and analyze available GHLM; specific attention will be paid to the following: underlying definitions and models, measured domains, adopted approaches (i.e., administration modes, such as performance-based approaches, and self-reported approaches), and assessment of SDH (i.e., whether the measure covered SDH) in the identified measurements.

## 2. Materials and Methods

A scoping review was conducted by following the five-stage approach developed by Arksey and O’ Malley [27]: identifying the research question; identifying relevant studies; study selection; charting the data to identify the key themes and concepts; and collating, summarizing, and reporting results from the selected studies.

### 2.1. Identifying the Research Question

The critical question that we aim to answer in this paper is, “What measurements are currently available to examine generic health literacy among adults?” Particular attention was paid to the four fundamental parts: (1) the underlying health literacy models and definitions; (2) the measured domains; (3) the adopted approaches; and (4) assessment of SDH.

### 2.2. Identifying Relevant Studies

The literature search was conducted to identify studies published from February 1990 to December 2019 in four electronic databases (Medline, EMBASE, Scopus, and Web of Science). The year of 1990 was chosen as the start date as the first health literacy tool was introduced in the early 1990s [28]. Boolean operators (AND/OR) were used to combine search terms in the search. The following search terms were used: first one was “health literacy”, the second one focused on the context of measurement (instruments* OR tool* OR questionnaire* OR survey* OR interview* OR assess* OR scale* OR measur* OR test* OR screen* OR psychometric*). The search algorithms of four databases were described in detail in Supplementary Materials S1.

### 2.3. Study Selection

**Figure 1:**
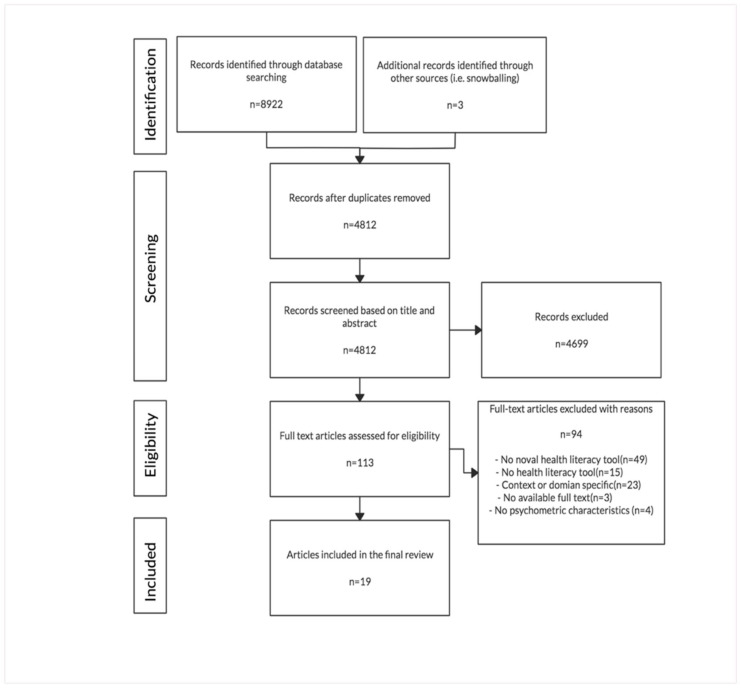
PRISMA Flow Diagram.

The studies were screened based on the Preferred Reporting Items for Systematic Review and Meta-Analysis (PRISM) flow diagram (Figure 1) [29]. The researcher (CYT) conducted the literature search on the four databases according to the search strategy in December 2019. Manual searches of the reference list of all included articles were also conducted to identify additional references. All the searched citations were stored in EndNote version X9 (Philadelphia, PA, USA; Clarivate).

The following inclusion criteria were used in the search: (1) peer-reviewed, published in English, and available in full text; (2) original publication describing the development or psychometric testing of generic health literacy measures; and (3) the age of target population ≧18 years. Studies which met the following criteria were excluded: (1) did not describe the development of the tool to measure health literacy; (2) described measurements assessing disease-specific or domain-specific health literacy; and (3) demonstrated the development of the modified measures of health literacy which were either modifications or short-form versions of original tools. The screening process was performed independently by two researchers (CYT and RHX), and any disagreement was resolved until consensus was achieved by our research team.

### 2.4. Data Charting and Collation

The characteristics of the identified measures, including authors, year of publication, measurements name, country developed in, research questions, theoretical foundation, validating sample, measured domains, items, administration time, and mode were extracted from the included studies by two researchers (CYT and RHX) independently. Consensus on the extracted data was reached by discussing it with the research team.

A domain (a.k.a. “component” or “dimension”) refers to the attribute or unobserved behavior that the research wants to measure in their study in health, social, and behavioral research [30]. In our study, we performed content analysis to identify the measured domains of health literacy. In most cases, the domains were labelled by the authors and explicitly explained in the original articles; we coded them as they were to keep the original interpretation. In terms of domains with same the meaning but different labels, we recoded them as the same label based on previous reviews of health literacy measurement tools and theoretical frameworks [17,18,19,31,32,33]. For example, we coded “quantitative” as “numeracy” which means the competencies to apply arithmetic operations and use numerical information; and coded “reading comprehension” as “prose” which refers to the abilities to read and understand text.

## 3. Results

The search process was summarized in the PRISMA flow chart (Figure 1). The searching strategy resulted in the initial identification of 8922 publications (Medline *n* = 1808, EMBASE *n* = 2767, Web of Science *n* = 3166, and Scopus *n* = 1181). A total of three additional articles were found by reference tracking and included in this review. After removing duplicates, 4812 articles remained, of which a further 4699 articles were excluded after screening the titles and abstracts. The full-text of the remaining 113 articles was reviewed and 94 articles were further excluded. Finally, 19 articles that report the generic health literacy instruments for adults remained.

### 3.1. Instrument Characteristics

Table 1 shows the characteristics of all identified measures (*n* = 19) assessing generic health literacy among adults. Most of the studies [34,35,36,37,38,39,40,41,42,43,44,45,46,47,48] (*n* = 17) were conducted in developed countries and regions (including the United States, European countries, Korean, Japan, and China (Taiwan)), few of them [49,50,51,52] (*n* = 2) were developed in developing counties (Iran and Thailand). The administration time of identified measurements ranges from 1 to 60 min. All identified measurements were analyzed from the aspects of underlying models and definition, measured domains, and adopted approaches. In terms of the assessment of SDH, we mainly examined whether it was addressed on the part of the underlying models and definition, and measured domains.

### 3.2. Underlying Definition and Models

The majority of studies [38,40,43,44,45,49,50,52] used the definition provided by Nutbeam [18], Ratzan and Parker [53], IOM [4], and Sørensen [19], which commonly addressed individuals’ abilities to access, understand, appraise, and apply health information and services that facilitate health decision making. For a few tools [34,35,36,48], the authors described different health literacy definitions, but did not mention which specific definition they used in the process of instrument development. According to the underlying models of the instruments, all instruments can be divided into the following three groups: (1) measurements summarized under Nutbeam’s conceptual model; (2) measurements summarized under Sørensen’s integrated model; and (3) measurements summarized under other models.

#### 3.2.1. Measurements Summarized under Nutbeam’s Conceptual Model

Among the identified measures, Nutbeam’s conceptual model [18] was most frequently used to guide the measurement development. Nutbeam mentioned three types of health literacy: functional, interactive, and critical health literacy [18]. Most of (*n* = 9) them [34,35,36,37,38,45,46,48,49] exclusively focus on the participants’ functional health literacy by examining their prose, numeracy, document, internet-based information seeking skills, and health knowledge. Only a few (*n* = 4) measures [40,44,50,52] assessed their critical health literacy and it mainly covered two essential components: the ability to evaluate the relevance and validity of health information, and to act politically to address social and economic determinants of health [18]. Only one measurement All Aspects of Health Literacy Scale (AAHLS) [40] involved the second component via assessing the subject’s empowerment at the level of community and social engagement. Results indicated that less measurements assess the ability to make beneficial decisions for their own health by modifying the SDH.

#### 3.2.2. Measurements Summarized under the Sørensen’s Integrated Model

By using the measurement model proposed by Sorensen and colleagues [19], the European Health Literacy Survey Questionnaire (HLS-EU-Q) [43] was developed to assess the individuals’ critical abilities of accessing, understanding, appraising, and applying health-related information across the domains of healthcare, disease prevention, and health promotion. Grounded in public health, this measurement not only addressed personal attributes to health literacy, but also reflected the interactive nature of health literacy by measuring the fit of personal’s abilities within contextual or situational demands of social systems [54]. In other words, this measure embraced SDH as an important component of its construct. However, the acknowledged limitation of this scale was that the majority of items generated from the Delphi process focused on the domain of healthcare and disease prevention, not in the domain of health promotion [43]. The domain of health promotion emphasized the capabilities to enable a citizen to access and process information on health determinants in the social and physical environment [43], whereas the domain of healthcare and disease prevention refers to a range of individual cognitive skills and capabilities applied in a medical context, not in a social realm. Enhancing the assessment of health promotion could be a possible way to improve the quality and applicability of this measurement in further research.

#### 3.2.3. Measurements Summarized under the Other Models

The five measurements (Swiss Health literacy Survey (HLS-CH), Health Literacy Management Scale (HeLMS), Health Literacy Questionnaire (HLQ), Iranian Health Literacy Questionnaire (IHLQ), and Health Literacy on Social Determinants of Health Questionnaire (HL-SDHQ)) were developed based on the authors’ conceptual framework aligned with relevant research questions. These measurements extended and supplemented the conceptualization of health literacy, especially from the perspective of SDH. The HLS-CH was developed to identify the shared core competencies of five patient-centered topics and identified “social roles” to address several SDH. These social determinants included the individuals’ public involvement in health policies, and knowledge of patients’ rights [39]. The HeLMS [41] and HLQ [42] were developed to capture the abilities that a person needs to have in order to get and use health information. The two measurements both considered “social support” as an essential component of health literacy skills by consulting or interviewing the participants’ experiences to look after his or her health, while the IHLQ addressed the factor “social empowerment” as an essential component of health literacy [51]. The HL-SDHQ was developed to evaluate the level of citizens’ abilities to understand the effect of SDH and the degree of implementation to take part in social health promotion activities [47].

### 3.3. Measured Domains of Health Literacy

**Figure 2:**
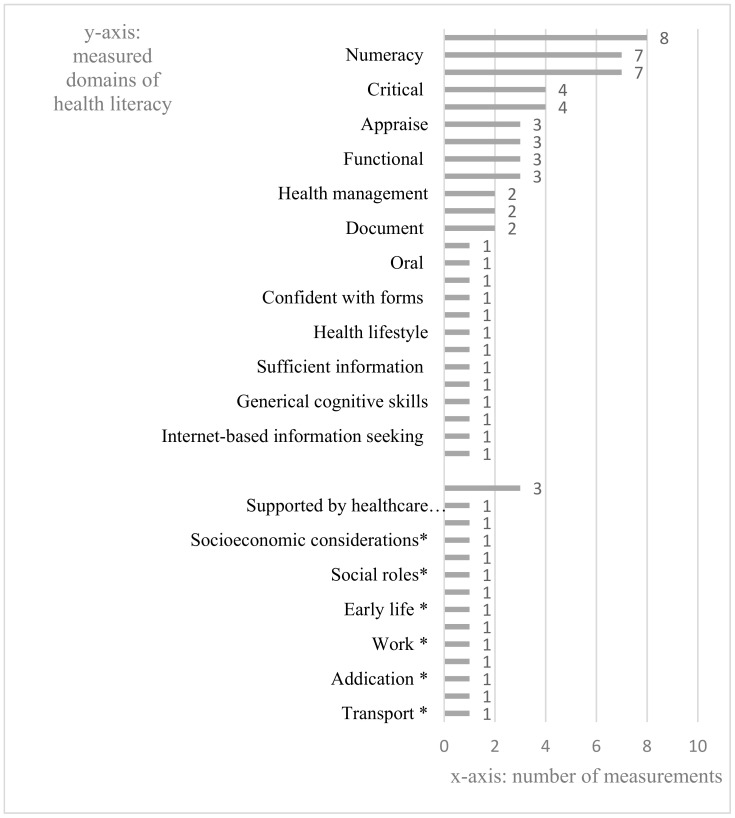
Measured Domains in the Identified Measurements. Notes: * = SDH-related domains; or non-SDH-related domains, otherwise.

In our content analysis, we found that certain studies [40,44,52] explicitly stated that their measurements assess subject’s functional, interactive, and critical health literacy. In this case, three domains were identified: “functional”, “interactive”, and “critical”. In Parker et.al [34], the authors indicated that their measures intended to assess reading comprehension and numeracy skills. According to our coding scheme, the “reading comprehension” was coded as “prose”. Hence, the domains “prose” and “numeracy” were identified. Overall, the content analysis highlighted a total of 39 different domains which were assessed in the 19 measurements (Figure 2). We used “The Solid Facts” which outlines the most important knowledge related to social determinants of health [55] as our main reference to categorize these domains. All domains were divided into two groups: SDH-related domains, and non-SDH-related domains.

Regarding SDH-related domains, we derived the following nine domains from “The Solid Facts”: “the social gradient”, “early life”, “social exclusion”, “work”, unemployment”, “social support”, “addiction”, “food”, and “transport”. In addition, we added the following five domains: “supported by healthcare providers”, “socioeconomic considerations”, “social capital”, “social empowerment”, “social roles”, which have the similar meaning with the domains highlighted in “The Solid Facts”, as SDH-related domains as well. A total of 14 domains were included in this group. The remaining identified domains were considered as the non-SDH-related domains. “Prose” was identified in eight measurements [34,36,38,45,46,48,49,51] and was the most prominent domain, followed by “numeracy” (*n* = 7 [34,36,38,45,46,48,49]), and “interactive” (*n* = 7 [40,41,42,44,50,51,52]). In the SDH-related group, “social support”, the most popular one, was only addressed in three measurements [41,42,47]; while all other domains were each assessed in only one measure. In summary, the majority of domains focused on the individual level factors related to health literacy, such as cognitive skills to access and process health information, and interactive abilities to express medical needs and make health appointment with health service providers. On the other hand, SDH including the social support and social capital were rarely assessed in the included health literacy instruments despite being highlighted as essential components of health literacy in the literature.

### 3.4. Adopted Approaches of Instruments

A total of eight identified measurements [34,36,37,38,45,46,48,49] used performance-based approaches to assess the participants’ actual health literacy skills by performing testing. The other 11 measures [35,39,40,41,42,43,44,47,50,51,52] used self-reported approaches to measure a participant’s perceived reading level, health information seeking skills, and beliefs. The performance-based measurements depended on an assessment format which was rooted in functional literacy measurements, examining the participants’ prose, numeracy, and document literacy. In contrast, the self-reported instruments address numerous domains of health literacy, such as individuals’ self-efficacy, perceived social support, self-reported social capital, and difficulties to access and process health care information.

## 4. Discussion

In the present study, after analyzing the essential parts of existing validated GHLM, we noticed that SDH has not been well-developed in the assessment. To advance the development of new GHLM, we further discussed the important role of SDH on health literacy measurements. We believe that the potential reasons why the majority of identified measures did not cover SDH comprehensively are the weak theoretical foundation, different constructs, and various subdomains of health literacy. Based on these identified issues, we also proposed some suggestions for the future development of new GHLM.

### 4.1. Importance of Social Determinants of Health to Health Literacy

In modern society, people are experiencing rapid spread of a large amount of valid and invalid information on the internet or other communication channels. To make an appropriate health-related decision, in the micro level, they need to critically examine the validity and reference of health information, ask suggestions from people who are trustworthy in their social network, and even make full use of the social resources they have. In the macro level, they are affected by the health system and cultural background within which they live. Moreover, scholars suggested that the final goal of the study to assess subjects’ health literacy level is not simply screening people and grouping them based on their health literacy level, but to identify the attributes that can be addressed to improve the people’s health literacy and then prove tangible benefits for their objective health status [26]. SDH are such kind of important attributes. Empirical research has highlighted that SDH like education level, socioeconomic status, physical limitations, and social empowerment were the factors with substantial contribution to inadequate health literacy [23,24]. It is argued that SDH should be covered in the measurements of generic health literacy.

### 4.2. Theoretical Foundation of Social Determinants of Health Literacy

In our study, we noted that the definition of health literacy mainly focuses on the individual’s capabilities to access, understand, appraise, and apply health information to make health-related decision. This skill-based definition of health literacy limited the development of validated instruments that can reflect the influence of SDH in this field. This finding is consistent with a previous review that the current literature have narrowly described health literacy as an individual-level factor [19,26]. It would be inadequate to only measure personal attributes but neglect the attributes of the social context in which the interaction takes place when they access and process health-related information and services. Facing such issue, some scholars suggest to consider health literacy as a relational concept that the abilities to use health-related information and services should be targeted to meet the requirements arising from the different social contexts in which the individual is embedded [56]. For example, the Health Literacy Network Germany and the German Network for Health Services Research proposed an updated definition of health literacy addressing the features of the educational, social, and health system that are prerequisites for individual [57]. By understanding health literacy from the perspective of relational sociology, most studies focused on the subjects’ abilities to leverage a social network to achieve health-related goals [58]. In these studies, several SDH, such as self-perceived support from families, friends, or significant others and self-reported social participation to facilitate health decision-making, were included in the actual measurements. These studies enriched the limited discussion of SDH on health literacy assessment. To conclude, conceptualizing health literacy as a relational concept may be an effective way to advance the assessment the influence of SDH on individuals’ health literacy skills.

### 4.3. Role of Social Determinants on the Constructs of Health Literacy

Health literacy is an evolving concept. It is not surprising that health literacy would include different constructs in different studies. Previous studies highlighted that a lack of consistent understanding of health literacy framework is a main weakness within the field [19,59,60]. In our review, we found that most of measurements were developed with a broad research question and without a specific focus on SDH. Lack of consensus on the theoretical model of health literacy contributed to the difficulties to precisely assess SDH as a component of the model. In terms of the assessment of SDH in the identified measurements’ framework, we noticed that the impact of SDH was acknowledged in the two most frequently used models: Nutbeam’s conceptual model and Sørensen’s integrated model, but remains unmentioned in many of the identified measurements. In addition, although several identified measurements supplemented the SDH in their frameworks, there were different operationalizations of the assessment of social determinants. For instance, while the HL-SDHQ [47] built the construct of health literacy with reference to the SDH, the framework of HLS-CH [39] was developed from more comprehensive perspectives covering the individual’s knowledge, health management skills, as well as SDH. In order to enhance the assessment of SDH of health literacy, researchers who develop health literacy measurements using Nutbeam’s or Sørensen’ model need to consider ways to improve the assessment of SDH. Specifically, it would be possible to further study the domain critical health literacy in Nutbeam’s model and the domain health promotion in Sørensen’s model which are closely associated with SDH but not fully discussed in current measurements. For researchers who work on developing a new GHLM based on their new models, it is also important to address the missing part of SDH and justify how it will differ from other existing measurements. Furthermore, it is noted that the construct of HL-SDHQ only covered SDH. It is deemed not adequate to develop a measure focusing exclusively on SDH as parts of health literacy. The new GHLM should be developed from a more comprehensive perspective covering both individual-level and social level attributes to health literacy. Overall, despite the models or frameworks adopted, the overall goal is to enhance health literacy measurement by using a systematic approach to provide clarity, precision, and transparency [61]. We believe that continuous exploration and efforts on health literacy will deepen the understanding of this topic.

### 4.4. Representative Social Determinants of Health Literacy

The effect of SDH is a broad area of research covering studies of how an individual’s social support, social capital, and social networks affect health behavior or community health. As this nature of SDH, the included measurements involved various approaches, focuses, and levels on this topic, posing difficulty in evaluating the actual impact of social determinants on health literacy. A research gap exists as to which SDH should be measured in a GHLM. Our study revealed that the interaction between perceived social support and health literacy was most frequently assessed. This finding is aligned with the result of Sentell and colleagues’ review of quantitative evidence in the field of health literacy, which found that the association of social support and health literacy has been most frequently assessed in empirical health literacy research [58]. For the other SDH identified in the present study, such as social capital which represents another increasingly important concept in health research, some scholars assumed that there is a trend towards measuring social capital across countries [62,63]. Although we only identified one study that assessed social capital in the health literacy context, we contended that it would be crucial to include social capital into the construct of health literacy in future measurements across different populations. Apart from the identified measurements covering SDH in this study, we can find other measures [64,65] which combined the health literacy items with SDH aspects from the database “Health Literacy Tool Shed [66]”. However, they still vary in the domains related to SDH, which indicated that a broad understanding of the interplay between individuals and their health-related environment existed. Under this situation, further research is needed to identify the representative indicators of social determinants on health literacy.

### 4.5. Adopted Approaches

We observed two main approaches to health literacy measurement. One is self-reported approach with a wide scope of measured dimensions, enabling a more in-depth and comprehensive operationalization of the construct “health literacy”. However, there is a concern about the accuracy of self-reporting measures, as the items tend to assess self-efficacy rather than health literacy in some cases [60]. Another is performance-based approach evaluating the individual’s actual ability of health literacy skills. Ethical issues related to the performance-based measure, however, existed as participants with limited health literacy skills may experience discomfort when they take part in the test and feel embarrassed by their poor abilities [67]. In addition to the two traditional approaches, other scholars found that health literacy was assessed by a mixed measurement approach [15]. Such an approach combines the methodological advantages of self-reported and performance-based approaches [15,68]. In terms of assessment of SDH of health literacy, the self-reported approach is needed to collect individual’s perceived social support from family members, friends, and health care providers, to explore the role of perceived social capital in processing health information and services, and to evaluate the influence of the individual’s social participation on their health literacy skills. Given the fundamental domains related to individual-level attributes of health literacy, a performance-based test would also be needed to deepen the understanding of the subject’s actual health literacy skills. Therefore, we argue that for further health literacy research, the combination of the two approaches can provide a valuable and effective way to generate a more detailed and precise assessment of generic health literacy in this field.

### 4.6. Limitations

There are some limitations in our study. First, we could not include all current available GHLM that may limit our analysis on this topic. On the one hand, although we followed the PRISMA guidelines to perform the scoping review, and used MESH terms and key words, some relevant literature might have been missed because of subjective judgements during the process. On the other hand, we only reviewed studies published in English; studies published in other languages have not been reviewed. Second, the qualities of the instruments were not assessed in our study since we conducted a scoping review. One acknowledged limitation of this method is a lack of evaluation of the quality of evidence [69]. In our study, we provided a descriptive summarization of identified measurements based on the collected information and proposed some suggestions for future measurement development. However, we could not give reference for the measurement with high credibility and validity. Third, the question items for some identified measurements were not available, which make it difficult to understand the true meaning of the domains and how the construct was operationalized. Therefore, we coded these domains as they were in order to avoid the false interpretation. As a result, some overlap between the coded domains existed, and it is hard to make precise categories for some domains. Fourth, it is not clear why a certain model covered SDH indexes at the conceptual level, while these indexes are missed at the operational level; how to appropriately address the assessment of SDH in the context of health literacy. SDH remains not well understood and faces particular challenges in its implementation. Even we noted that a plethora of health literacy measurements existed and some of them covered SDH from “Health Literacy Tool Shed”, further study is needed to enhance the insights of the interaction between SDH and health literacy.

## 5. Conclusions

Our review found that although current measurements of generic health literacy adopted a multidimensional construct, they mainly focused on individuals’ abilities to access and apply health resources. Furthermore, the interplay between SDH and health literacy has not been well understood in the measures. Currently, measuring SDH factors in the context of health literacy are gaining importance in terms of their substantial contribution to inadequate health literacy. The weak theoretical foundation, different constructs, and various subdomains on health literacy may cause that SDH has not been measured in a comprehensive way. Specifically, we noted that (a) the majority of definition of health literacy focus on the individual’s level instead of the social level; (b) the impact of SDH on health literacy was addressed on the most adopted models: Nutbeam’s conceptual model and Sørensen’s integrated model, but were not fully implemented in their instruments; (c) from the measured domains perspective, there is no consensus on representative subdomains related to SDH. Given the importance and omission of SDH on this topic, further research is required to advance the assessment of the interaction between SDH and health literacy, and to eventually develop a new reliable and validated GHLM. Conducting the survey of generic health literacy is an essential population-based way to identify people with limited health literacy and develop interventions to advance population health literacy. Therefore, addressing the above issues is the prerequisite for conducting a GHLM in the community. We believe a new GHLM covering SDH can help us to have a better understanding of the population health literacy level and create a health literate environment by modifying the SDH.

## Figures and Tables

**Table 1:** Instrument characteristics of tools assessing generic health literacy among adults.

Scale Names	Year Nation	Research Questions	Theoretical Basis	Validated Sample	Domains, Items (#)	Domains	Administration Mode and Time
The Test of Functional Health Literacy in Adults [34] (TOFHLA; Parker, R.M., Baker, D.W., et al.)	1995 US	To develop a valid and reliable instrument to measure the functional health literacy for patients.	Not mentioned	200 participants (18+ years)	2, 67	(1) Prose (reading comprehension); (2) Numeracy (competencies to apply arithmetic operations and use numerical information).	Performance-based 22 min
Brief Health Literacy Screener [35] (BHLS; Chew, L.D., Bradley, K.A., et al.)	2004 US	To develop a screening tool for identifying patients with inadequate or marginal health literacy.	Not mentioned	332 participants (18+ years)	3, 3	(1) Help read; (2) Confident with forms; (3) Problems learning (about medical condition because of difficulty understanding written information).	Self-reported 1 min
The Newest Vital Sign [36] (NVS; Weiss. B.D., Mays, M.Z., et al.)	2005 US	To develop a quick and accurate screening tool for the individuals’ with inadequate literacy.	Not mentioned	500 participants (18+ years)	2, 6	(1) Prose; (2) Numeracy.	Performance-based 3 min
The Public Health Literacy Knowledge [37] Scale(PHLKS; Pleasant, A., Kuruvilla, S.)	2008 US	To develop a valid and reliable public health literacy knowledge sale at the population level.	Essential facts for life messages	829 participants (Average age = 37 years)	1, 17	(1) Health knowledge.	Performance-based Not mentioned
Korean Health Literacy Scale [48] (KHLS; Lee, T.W., Kang, S.J., et al.)	2009 KR	To develop a valid and reliable screening test for limited health literacy for older Korean adults.	Not mentioned.	411 community dwelling (60+ years)	3, 24	(1) Prose; (2) Numeracy; (3) Health knowledge.	Performance-based 15–20 min
Health Literacy Skills Instrument [38] (HLSI; McCormack, L., Bann, C., et al.)	2010 US	To develop a comprehensive and skill-based instrument to measure an individual’s health literacy.	Ratzan and Parker’s definition of health literacy.	889 participants (18+ years)	5, 25	(1) Prose; (2) Document; (3) Numeracy; (4) Oral (listening or audiovisual); (5) Internet-based information seeking.	Performance-based (computer-based) 45 min
Mandarin Health Literacy Scale [49] (MHLS; Tsai, T., Lee, S.Y.D, et al.)	2010 China (Taiwan)	To develop a culturally suitable screening test for people who speak Mandarin Chinese.	IOM’s definition of health literacy.	448 individuals (18+ years)	3, 50	(1) Prose; (2) Document; (3) Numeracy.	Performance-based Not mentioned
Swiss Health Literacy Survey [39] (HLS-CH; Wang, J., Thombs, B.D., et al.)	2012 (CH)	To identify specific capabilities for health in definitions of health literacy and patient-centered concepts among the general population.	Developed a broad, inclusive framework by focusing on the level of shared core competencies of five patient-centered topics.	1250 respondents (15+ years)	5, 73	(1) Information and knowledge; (2) General cognitive skills (literacy, numeracy and self-expression, interpersonal, problem-solving, critical decision-making); (3) Social roles (address social determinants of health, and consumer competencies); (4) Health management; (5) Health lifestyle.	Self-reported (telephone interview and face-to-face interview) 30 min
All Aspects of Health Literacy Scale [40] (AAHLS; Chinn, D., MaCarthy, C.)	2013 UK	To develop a tool to measure functional, communicative, and critical health literacy in primary healthcare setting.	Nutbeam’s definition and conceptual model.	146 participants (15–82 years)	3, 14	(1) Functional; (2) Interactive; (3) Critical.	Self-reported 7 min
Health Literacy Management Scale [41] (HeLMS; Jordan, J.E., Buchbinder, R. et al.)	2013 AUS	To develop a tool to assess health literacy constructs crucial to patients when accessing, understanding, and using health information within the health systems.	Developed a conceptual framework of health literacy from the perspective of patients.	350 participants (40+ years)	8, 29	(1) Attitudes; (2) Understand; (3) Social support; (4) Socioeconomic considerations; (5) Assess; (6) Interactive; (7) Being proactive(take proactive steps to source and understand health information to better address a health issue); (8) Apply.	Self-reported No time limit
Health Literacy Questionnaire [42] (HLQ; Osborne, R.H., Batterham, R.W., et al.)	2013 AUS	To develop an instrument to measure health literacy needs and challenges across individuals, organizations.	Developed a comprehensive model covered the full range of health literacy capabilities in a real-world setting from the perspective of general population, practitioners, and policy makers.	405 participants (18+ years)	9, 44	(1) Supported by healthcare providers; (2) Sufficient information; (3) Health management; (4) Social support; (5) Appraise; (6) Interactive; (7) Navigation; (8) Access; (9) Understand.	Self-reported No time limit
European Health Literacy Survey Questionnaire [43] (HLS-EU-Q; Sørensen, K., Broucke, S.V., et al.)	2013 EU	To develop a valid and reliable tool to measure the comprehensive construct of health literacy in different populations.	Sørensen’s definition and conceptual model of health literacy.	99 participants (15–81 years)	4, 47	(1) Access; (2) Understand; (3) Appraise; (4) Apply.	Self-reported 20–30 min
Korean Health Literacy Instrument [44] (KHLI; Kang, S.J., Lee, T.W., et al.)	2014 KR	To develop and validate an instrument measuring the ability to understand and use health-related information and make informed health decisions in Korean adults.	Nutbeam’s definition and conceptual model.	315 participants (40–64 years)	3, 18	(1) Functional; (2) Interactive; (3) Critical.	Self-reported 25 min
Japanese Functional Health Literacy Test [45] (JFHLT; Nakagami, K., Yamauchi. T., et al.)	2014 Japan	To develop a reliable and valid tool of functional health literacy in a Japanese clinical setting.	Function health literacy.	535 Japanese outpatients (22+ years)	2, 16	(1) Prose; (2) Numeracy.	Performance-based 10–15 min
Comprehensive Health Activities Scale [46] (CHAS; Curtis, L.M., Revelle, W., et al.)	2015 US	To develop a comprehensive tasks-based health literacy measurement.	Developed a conceptual framework covered nine scenarios. depicting health-related tasks that patients often experience.	826 participants (55–74 years)	2, 45	(1) Prose; (2) Numeracy.	Performance-based 60 min
Taiwanese Health Literacy Assessment Tool [50] (THLAT; Chung, M.H., Chen, L.K., et al.)	2015 China (Taiwan)	To evaluate health literacy among urban elderly in Taiwan.	Nutbeam’s definition and conceptual model	1082 elderly (60+ years)	2, 10	(1) Interactive; (2) Critical.	Self-reported 3–5 min
Iranian Health Literacy Questionnaire [51] (IHLQ; Haghdoost, A.A., Rakhshani, F., et al.)	2015 Iran	To develop a valid and reliable instrument to measure and monitor community health literacy in Iran.	Develop a framework to measure health literacy in exposure to disease and health promotion approach adjusted for the Iranian culture.	1080 participants (18–60 years)	7, 36	(1) Prose; (2) Individual empowerment (household medical equipment use); (3) Interactive; (4) Access; (5) Appraise; (6) Social empowerment; (7) Health knowledge.	Self-reported No time limit
ABCDE Health literacy scale [52] (ABCDE-HLS; Intarakamhang, U., Y. Kwanchuen)	2016 Thai	To develop an instrument to measure health literacy based on the concept of ABCDE (alcohol, baccy, coping, diet, and exercise) behavior for risk reduction	Nutbeam’s conceptual model.	4401 people (15+ years)	3, 64	(1) Functional; (2) Interactive; (3) Critical.	Self-reported No time limit
Health Literacy on Social Determinants of Health Questionnaire [47] (HL-SDHQ; M. Matsumoto, K. Nakayama)	2017 Japan	To develop a tool measuring health literacy from the perspective of social determinants of health.	Sorensen’s definition of health literacy for the domain of health promotion; the solid fact developed by CSDH.	831 adults (18+ years)	10, 33	(1) Social gradient; (2) Early life (the health impact of early development and education lasts a lifetime); (3) Social exclusion; (4) Work; (5) Unemployment; (6) Social support; (7) Social capital; (8) Addiction (9) Food; (10) Transport.	Self-reported No time limit

Notes: KR = Korean; CH = Switzerland; CSDH = The World Health Organization Commission on the Social Determinants of Health; # = number of domains and items.

## Supplementary Materials

The following are available online at https://www.mdpi.com/1660-4601/17/21/7768/s1, Supplementary Materials S1: Searching methodology.
