# Supplementary material for: Generic Health Literacy Measurements for Adults: A Scoping Review
Source: Int J Environ Res Public Health. 2020 Oct 23;17(21):7768. doi: 10.3390/ijerph17217768 (PMC7660647; doi:10.3390/ijerph17217768)
Supplement: Supplementary file 1 [file ijerph-17-07768-s001.pdf]

⚜ Medline

Search results: *n* =1808
Search algorithm:

1. (health adj literacy).ti,ab.
2. (scale* or instrument* or tool* or questionnaire* or survey* or screen* or interview* or measure* or psychomet* or assess*).ti,ab.
3. (develop* or validat* or psychomet* or factor analysis* or reliability* or validity*).ti,ab.
4. 1 and 2 and 3
5. (review or systematic review or meta-analysis or scoping review).pt.
6. 4 not 5
7. limit 6 to (english language and yr="1990 -Current")

⚜ Embase

Search results: *n* = 2767
Search algorithm:

1. (health adj literacy).ti,ab.
2. (scale* or instrument* or tool* or questionnaire* or survey* or screen* or interview* or measure* or psychomet* or assess*).ti,ab.
3. (develop* or validat* or psychomet* or factor analysis* or reliability* or validity*).ti,ab.
4. 1 and 2 and 3
5. (review or systematic review or meta-analysis or scoping review).pt.
6. 4 not 5
7. limit 6 to (english language and yr="1990 -Current")

⚜ Web of Science

Search results: *n* = 3166
Search algorithm

# 4	3,166	**(#3) *AND* LANGUAGE: (English)**<br>*Indexes=SCI-EXPANDED, SSCI, A&HCI, CPCI-S, CPCI-SSH, BKCI-S, BKCI-SSH, ESCI*<br>*Timespan=1990-2020*
# 3	3,255	#1 NOT #2<br>*Indexes=SCI-EXPANDED, SSCI, A&HCI, CPCI-S, CPCI-SSH, BKCI-S, BKCI-SSH, ESCI*<br>*Timespan=All years*
# 2	1,946,387	DT=(review OR systematic review OR meta-analysis OR scoping review)<br>*Indexes=SCI-EXPANDED, SSCI, A&HCI, CPCI-S, CPCI-SSH, BKCI-S, BKCI-SSH, ESCI*<br>*Timespan=All years*
# 1	3,478	TS=(("health literacy") AND (scale* OR instrument* OR tool* OR questionnaire* OR survey* OR screen* OR interview* OR measure* OR psychomet* OR assess*) AND (develop* OR validat* OR psychomet* OR factor analysis* OR reliability* OR validity*))

		Indexes=SCI-EXPANDED, SSCI, A&HCI, CPCI-S, CPCI-SSH, BKCI-S, BKCI-SSH, ESCI Timespan=All years

⚜ **Scopus**

Search results: *n* =1181

Search algorithm:

TITLE-ABS ((health AND literacy) AND (scale* OR instrument* OR tool* OR questionnaire* OR survey* OR screen* OR interview* OR measure* OR psychomet* OR assess*) AND (develop* OR validat* OR psychomet* OR factor AND analysis* OR reliability* OR validity*)) AND (EXCLUDE (DOCTYPE , "re")) AND (EXCLUDE (PUBYEAR , 1989) OR EXCLUDE (PUBYEAR , 1988) OR EXCLUDE (PUBYEAR , 1986) OR EXCLUDE (PUBYEAR ,1985) OR EXCLUDE (PUBYEAR ,1982) OR EXCLUDE (PUBYEAR , 1980) OR EXCLUDE (PUBYEAR, 1977) OR EXCLUDE (PUBYEAR, 1974)) AND (LIMIT-TO (ACCESSTYPE(OA))) AND (LIMIT-TO (LANGUAGE , "English")).
